# Highly innovative drugs in the Czech Republic: retrospective analysis of impact on early market entry, regular reimbursement system entry and public pharmaceutical expenditure

**DOI:** 10.1186/2052-3211-8-S1-O3

**Published:** 2015-10-05

**Authors:** Anna Lukačišinová, Helena Skácelová

**Affiliations:** 1 State Institute for Drug Control (SIDC), Prague, 100 41, Czech Republic

## Background

Growing unmet need in treatment of very severe and rare diseases is enhancing a development of new innovative drugs. Consequently, the inclusion of these drugs in the reimbursement system is becoming a critical issue in many countries, as most of them prove their clinical and cost effectiveness just partly at the time of submission.

## Objectives

This study aims to describe a specific system of reimbursement of highly innovative drugs (HID) in the Czech Republic, representing a time-limited reimbursement under special conditions. The second objective is to evaluate the total number of drugs approved as HID, as well as the number of drugs entering or failing to enter the system of regular reimbursement. The third objective is to describe the development of costs and impact of HID on the overall public pharmaceutical expenditures.

## Methods

This is a retrospective observational study. Data for HID submissions and approvals were searched for 2008-2015. Data were analysed in order to capture the main characteristics of drugs (ATC code, indication). The length of stay in the temporary reimbursement and reasons for non-acceptance/acceptance into the regular reimbursement system were analysed for each drug individually. The overall costs of HID covered by health care payers were evaluated for 2009-2014.

## Results

There were 52 drugs approved as HID during 2008-2015, with 21 already having entered the system of regular reimbursement. Most of the drugs belong to the group of antineoplastic agents. The highest number of submissions was reported in 2011 with 13 submissions, while 10 of these drugs succeeded to enter the regular system. Since 2009 expenses of HID doubled from 36 to 79 million Euro/year in 2014, while the overall out-patient expenditures on drugs remained stable (1,834-1,862 million Euro/year).

## Conclusions

Reimbursement of drugs in the specific system of HID represents a way of early introduction to the market while giving an opportunity to deal with unmet need and the time for proving clinical and cost effectiveness. Limitations of the system are driven mainly by insufficient data from real clinical practice at the end of the limited time frame and subsequent inability to prove cost effectiveness in clinical practice.

## Lessons learned

Despite some disadvantages, the system of HID is a good tool to provide treatment of very severe and rare diseases.

